# Comment on Huang et al. Accuracy and Agreement of a 45 mg Versus a 75 mg ^13^C-Urea Breath Test for the Diagnosis of *Helicobacter pylori* Infection in Adults: A Randomized Crossover Trial. *Diagnostics* 2026, *16*, 1567

**DOI:** 10.3390/diagnostics16182922

**Published:** 2026-09-10

**Authors:** Agostino Di Ciaula, Piero Portincasa

**Affiliations:** Clinica Medica ‘A. Murri’, Department of Precision and Regenerative Medicine and Ionian Area (DiMePre-J), University of Bari ‘Aldo Moro’ Medical School, Piazza Giulio Cesare, 11, 70124 Bari, Italy; piero.portincasa@uniba.it

In patients who do not require endoscopic evaluation, assessment for active *H. pylori* infection relies on accurate, noninvasive, nonserologic tests, such as the urea breath test (UBT). Patients drink a citric acid solution containing urea labelled with the non-radioactive carbon isotope ^13^C. Hydrolysis of urea by *H. pylori* urease produces labelled CO_2_ allowing *H. pylori* infection to be detected by measuring ^13^C concentrations in breath samples. The sensitivity and specificity of UBT are both approximately 95% [1].

In this context, we read with great interest the recent article by Huang et al. reporting substantial agreement and comparable diagnostic performance between a 45 mg and a 75 mg ^13^C-urea breath test (^13^C-UBT) for the diagnosis of *Helicobacter pylori* infection [2]. The authors addressed an important topic, with potential implications for cost reduction and simplification of ^13^C-UBT protocols.

Nevertheless, several methodological issues deserve further consideration before accepting the conclusion that the 45 mg protocol is clinically comparable to the standard 75 mg protocol.

The diagnostic accuracy analysis relies on a composite reference standard that assumes concordant results between the two ^13^C-UBT protocols represent the true infection status, while only discordant cases undergo endoscopic verification. This approach introduces a substantial risk of incorporation bias and circular reasoning because the index tests themselves contribute to the definition of the reference standard. Consequently, estimates of sensitivity, specificity, predictive values, and area under curve (AUC), as well as claims of non-inferiority may be systematically inflated. Although the authors appropriately acknowledge this limitation, its potential impact on the validity of the diagnostic performance estimates may be greater than suggested.

In addition, the study should not be interpreted as a pure dose-comparison trial. First, no information is provided regarding the manufacturer of the two different substrates. Moreover, the 45 mg protocol used a tablet formulation administered without citric acid, whereas the 75 mg protocol used a granule formulation containing citric acid. This approach generates a questionable combined and simultaneous variation of substrate dose, formulation, and gastric acidification. Indeed, gastric acidity decreases intragastric pH, suppresses non-*H. pylori* urease activity, and delays gastric emptying [3]. These effects may promote more uniform intragastric distribution of urea, and prolong its contact with *H. pylori* urease, thereby affecting the urease-substrate interaction [4], and potentially increasing mean ^13^C-UBT values compared with the control group [5]. An additional mechanism involves the direct effects of ascorbic acid and citric acid on, or their effects on the accessibility of, the postulated urea channel (i.e., UreI) thereby enhancing urea permeability [6]. Based on these properties and the available evidence, the Maastricht VI/Florence Consensus Report strongly endorses the use of citric acid as an essential component of the ^13^C-UBT [7]. This evidence also appears to apply to Asian populations, as a Chinese cohort study found that citric acid enhanced the accuracy of ^13^C-UBT for the diagnosis of *H. pylori* infection [8]. Thus, the differences observed by the authors between the 45 mg and 75 mg protocols cannot be attributed solely to the amount of ^13^C-urea administered per se.

Further methodological concerns arise from performing both ^13^C-UBTs on the same day, separated by an average interval of approximately 153 min. Although each test included a new baseline breath sample, the possibility of residual isotope effects cannot be completely excluded. Additional information would be valuable, including baseline delta-over-baseline (DOB) values before the second test, DOB distributions according to randomization sequence, and a formal assessment of carryover effects comparing the 45 mg followed by 75 mg and 75 mg followed by 45 mg sequences. Such analyses would strengthen confidence that the crossover design did not influence results.

The selection of diagnostic cutoffs also warrants further scrutiny. The reported thresholds (>3.9‰ for the 45 mg protocol and >5.1‰ for the 75 mg protocol) were derived by optimizing the Youden index. Because these cutoffs were both generated and evaluated in the same dataset and under the same composite reference standard, there is a risk of overestimating diagnostic performance. Sensitivity analyses using predefined and clinically validated cutoff values would help determine whether the reported findings remain robust under conditions that more closely reflect routine clinical practice.

Another relevant point is that the study population included both treatment-naïve individuals and patients undergoing post-eradication testing. These groups may differ in bacterial density, urease activity, and expected DOB values. Importantly, post-eradication testing represents a clinical setting in which lower-dose protocols may be particularly vulnerable to false-negative results because of reduced bacterial burden and borderline DOB values. Separate diagnostic accuracy analyses for these two indications would therefore be highly informative.

Finally, the analysis of the 42 discordant cases deserves careful interpretation. Although these data provide useful insights, they originate from a selected subgroup and should not be used to infer overall test performance. Nonetheless, the finding that the 75 mg protocol correctly identified a substantially higher proportion of endoscopically confirmed positive cases raises the possibility that the lower-dose protocol may be less sensitive in borderline infections. Rather than providing evidence of equivalence, these discordant cases may highlight residual uncertainty regarding the interchangeability of the two protocols and support the need for additional validation studies employing an independent reference standard (e.g., endoscopy) in all participants.

In conclusion, based on the available evidence, the diagnostic accuracy of the ^13^C-UBT may depend only partly on the dose of ^13^C-urea administered. Other factors, such as the administration of citric acid and the clinical features of patients (i.e., ethnicity, treatment-naïve individuals or post-eradication confirmation, possible different bacterial density and/or urease activity) may also play a critical role. In addition, comparing two different protocols should consider a solid methodological approach, also taking into account an adequate reference standard, possible carryover effects, the use of standardized cutoffs. The study by Huang et al. [2] provides valuable preliminary evidence that a 45 mg ^13^C-UBT protocol may achieve substantial agreement with a standard 75 mg protocol. However, the use of a composite reference standard, simultaneous changes in dose and formulation, the absence of a formal carryover analysis, optimization-derived cutoffs, the inclusion of heterogeneous clinical populations, and the interpretation of discordant cases all warrant cautious interpretation. Further studies designed to minimize potential confounders, to use independent reference standards and externally validated diagnostic thresholds, and to enroll homogeneous populations will be necessary before widespread implementation of the lower-dose protocol can be recommended.
